# Vibrational resonance in feed-forward-loop neuronal network motifs

**DOI:** 10.1186/1471-2202-16-S1-P189

**Published:** 2015-12-18

**Authors:** Ali Calim, Ugur Ileri, Muhammet Uzuntarla, Mahmut Ozer

**Affiliations:** 1 Department of Biomedical Engineering, Bulent Ecevit University, Zonguldak 67100, Turkey; 2 Department of Electrical and Electronics Engineering, Bulent Ecevit University, Zonguldak 67100, Turkey

## 

A neuron carries out its functions in networks receiving contacts from roughly 10^4 ^presynaptic neurons. Such a dense connectivity profile for a single neuron may give rise to enormous complex neuronal topologies, which might be very difficult to understand the underlying mechanisms of neurological functions and diseases. Extensive experimental data from neuroanatomical studies have uncovered that neural networks include some recurring topologies of microcircuits, known as *network motifs *which serve as characteristic building blocks of complex networks [1,2]. Therefore, it is widely assumed that a clear explanation on dynamical and functional features of these network motifs can be considered as the first step to understand large networks.

**Figure 1:**
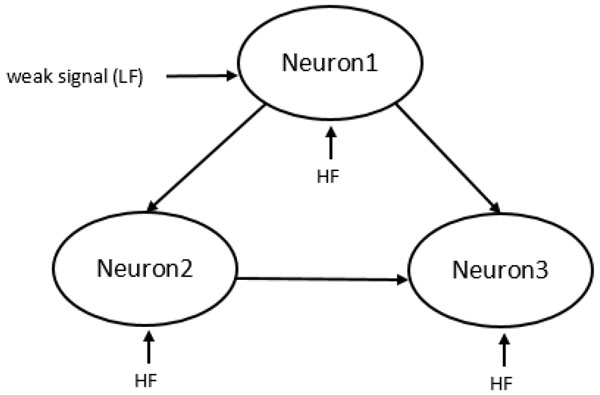
**Schematic illustration of the considered feed-forward-loop motif**. LF and HF refers to low and high frequency signals, respectively. Neuron 1 and 3 is considered as input and output neurons, respectively.

Following this motivation, in this study, we investigate the Vibrational Resonance (VR) phenomenon in a triple-neuron feed-forward-loop (FFL) which is one of the most significant brain network motifs, shown in Figure 1. VR is a physical phenomenon found in nonlinear systems where a weak signal can be detected and processed by the system with the assistance of another high frequency signal. It is very similar to the well-known stochastic resonance phenomenon, where the role of noise is replaced in VR by a high frequency signal. In recent years, there exists a growing interest in applications of VR to neuroscience because two frequency signals are pervasive in neural systems, i.e. bursting neurons exhibit two widely different time scales, simultaneous arrival of vocal signals having distinct frequencies to auditory neurons. Due to the significance of its potential in neural signal processing, the VR phenomenon has already been studied with computational models of neurons and their networks [3,4]. However, a population of excitatory and inhibitory neurons has not been considered, yet. Here, we study the VR dynamics depending on whether the neurons in the FFL motifs are excitatory or inhibitory considering eight possible structural configurations of the considered microcircuit (Table 1). Such an approach provides us to test the functional influences of structural configuration on VR dynamics in neural populations.

**Table 1:** Eight possible FFL types.

Type	Neuron 1	Neuron 2	Neuron 3
T1-FFL	E	E	E
T2-FFL	E	I	E
T3-FFL	E	E	I
T4-FFL	E	I	I
T5-FFL	I	E	E
T6-FFL	I	I	E
T7-FFL	I	E	I
T8-FFL	I	I	I

E and I refer to excitatory and inhibitory neurons, respectively.

We found that the weak signal transmission in the network via the VR mechanism is possible depending on both the coupling strength between neurons and the network structure. For instance, when a weak coupling strength is present between connected neurons, weak signal injected to the input neuron can be transmitted to the output with only T8-FFL motif. In this case, signal transmission is impossible for other types of network motifs. For intermediate coupling strengths, signal transmission performance is high for all motifs and motif type does not change very much VR dynamics. Finally, for strong coupling strengths, the best VR performance is obtained with T1-FFL motif where all the neurons in the network are excitatory. We also clarify the mechanisms that underlies the differences in performance of network motifs.
